# Effect of medial stabilizer chest position on pectus bar dislocation

**DOI:** 10.1007/s00383-024-05822-w

**Published:** 2024-08-18

**Authors:** Caroline Melhado, Alexandra Highet, Neal Mukherjee, Doruk Ozgediz, Olajire Idowu, Sunghoon Kim

**Affiliations:** 1 Department of Surgery, University of California San Francisco, San Francisco, CA USA; 2 Division of Pediatric Surgery, University of California San Francisco Benioff Children’s Hospitals, 744 52nd Street, Oakland and San Francisco, CA 94609 USA

**Keywords:** Pectus excavatum, Stabilizer, Bar dislocation, Return to full activity, Degrees of freedom

## Abstract

**Purpose:**

The current standard method for pectus excavatum (PE) repair is the Nuss procedure. One major postoperative complication is the displacement of the implanted metal bar, which is used to remodel the chest wall. Blocking the possible ways that the bar can be displaced with the use of stabilizers and peri/intracostal sutures has reduced the incidence of bar displacement. Despite the modifications, bar dislocation is often reported. We adopted the medial position stabilizer placement method and imposed no postoperative restrictions. In this study, we analyzed the bar dislocation rate with this modification and concurrent postoperative full activity.

**Methods:**

Nuss procedure modification where stabilizers are placed bilaterally in the medial location was done on patients irrespective of age and Haller index greater than 3.25. A single bar was used for all patients. Cryoanalgesia was performed on every patient. No postoperative restrictions were imposed on the patients. Full immediate activities, including sports, were allowed.

**Results:**

114 patients (103 male, 11 female) were analyzed from 2016 to 2023. The median age was 15 years old. There was zero incidence of bar displacement. The combined incidence of other postoperative complications was 4%: 2 wound infections and 2 hematoma formations, both needing incision and drainage.

**Conclusion:**

Bilateral medial stabilizer placement resulted in no incidence of bar dislocation. Return to immediate full activities after the Nuss procedure did not appear to increase the incidence of bar displacement if stabilizers were placed medially.

## Introduction

Pectus excavatum is the most common chest wall deformity repaired surgically. The Nuss procedure is the current preferred approach. One of the well-known and dreaded complications of the operation is bar displacement [1]. To discuss the causes of bar displacement, the degree of freedom of an object needs to be considered. Any object, including a metal bar, floating in space has six degrees of freedom (DOF) [2]. Once the bar is inserted into the chest using the Nuss procedure, the DOF for the bar decreases to three: the metal bar can shift from its original position by sliding laterally along the left-right (frontal) axis, rotate around the frontal axis, or sink dorsally along the anteroposterior (sagittal) axis. These dislocations usually necessitate a reoperation [3]. Over the past 30 years, various techniques have been proposed to decrease the incidence of bar displacement. Introduction of new techniques (eg., medial positioning of stabilizer [4, 5]), additional tools, and accessories such as stabilizers, peri/intracostal suturing, claw fixation, bridge bar fixation, and multiple bar insertion has been reported in the literature, with bar displacement rates between 0 and 5% [1, 4–12]. Specific implantation techniques vary by surgeons, and currently, there is no consensus on the optimal technique [7]. This is partly due to the inaccessibility and variability of metal bars and stabilizer/accessory implants that are available to surgeons in different regions of the world. In the United States, metal bar and stabilizer designs have been unchanged due to the patent rights, which expired in 2018, held by a single company for many years. Over time, different implant designs are becoming available in the USA and being imported from abroad. To block the three DOFs of an implanted metal bar, we surmised bilateral medial placement of stabilizers should block all three DOFs.

There are concerns that early postoperative activities and sports may increase the chances of bar displacement. It is a frequent practice to recommend very conservative postoperative activity restrictions to patients, such as several weeks at home before returning to school and several months before resuming sports, if at all [7–9]. However, data to support the benefits of these restrictions is lacking, and there are no evidence-based clinical practice guidelines for post-Nuss procedure activity. So, it is unclear whether such limitations are necessary [9]. For this study, we authorized the patients to resume any non-contact exercise postoperative day 1, although we expected that most patients would not be able to due to chest stiffness even with cryoanalgesia. Contact sports could resume 2 weeks after the operation. This was done to impress upon them that they needed to mobilize and begin strength training as soon as they could.

For this study, we aimed to review the rate of bar displacement when using a technique employing bilateral medial stabilizer placements, which inhibits bar displacement in all 3DOF and allows immediate return to full activity. Our center’s technique has been previously discussed as related to the elevation of the chest using the T-suture technique for safer dissection of the anterior mediastinum, management of asymmetric pectus excavatum, and cryoanalgesia which was instituted in 2016 [13–15].

## Methods

### Analysis

**Fig. 1:**
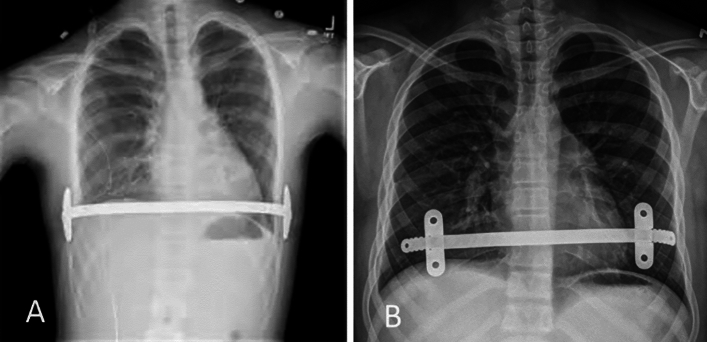
The medial position of the stabilizer is defined as when the chest is viewed from anterior to posterior direction; the holes located in the stabilizers can be discerned as a circle. **A** The holes on the stabilizers are not visible. **B** The holes on the stabilizers are clearly seen as circles

We conducted a retrospective cohort study of all children who underwent Nuss bar placement at our institution over an 8 year period (Jan 2016-Jan 2023) using bilateral medially positioned stabilizers. The length of the metal bars used tended to be on the shorter side; on average, a 9-to-10 inch length was used. The medial position is defined as the holes in the stabilizer can be seen as a circle, not an ellipse, on an anteroposterior chest X-ray (Fig. 1). We collected patient characteristics, inpatient interventions, postoperative complications, and bar/stabilizer removal complications via chart review. Length of hospital stay was defined as the period starting after the operation. Bar stability assessment was based on physical examination during follow-up visits. The incidence of bar migration or displacement was based on physical examination (visual inspection) and counting cases where surgical correction was needed. Follow-up was defined as every 6 months postoperatively until bar removal. The Institutional Review Board of the University of California San Francisco approved the study. The analysis was completed using Stata, version 14.0 (StataCorp, LLC, College Park, TX).

### Operative technique

**Fig. 2:**
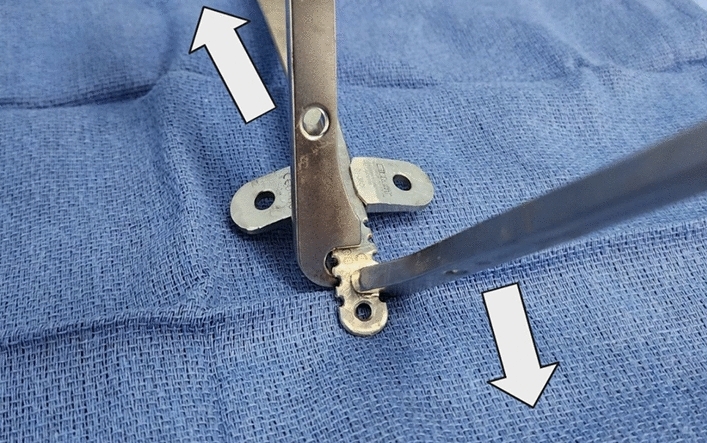
After placing the stabilizer in the medial position, the end of the metal bar is bent with Synthes bar benders

**Fig. 3:**
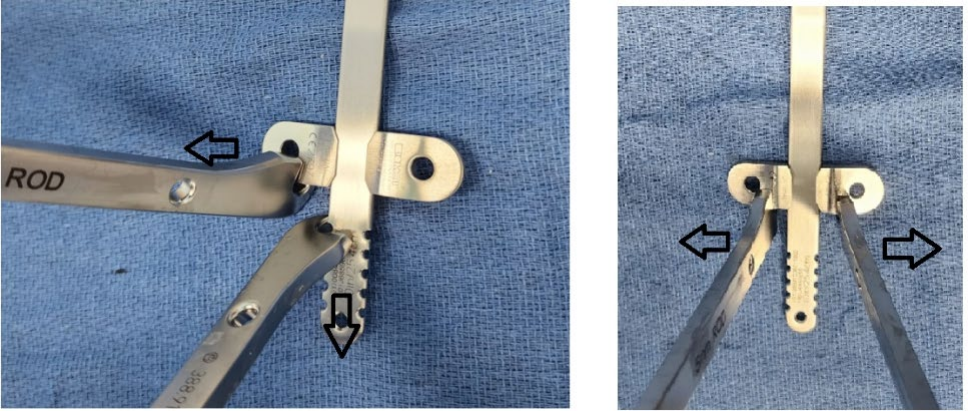
Synthes bar benders are used to disengage the stabilizer from the metal bar at the time of the metal bar removal. Arrows indicate the direction of force applied to bend the stabilizer off the metal implant

Three surgeons conducted the Nuss bar placement for all participants in the study using the same operative technique [14]. The procedure begins with a bilateral transverse anteriorized lateral chest incision followed by a 5 mm port placement through the same incision wound on each side. A 25-gauge needle was inserted near the sternum staying lateral to the mammary vessels with videoscope observation. Once a safe medial position is identified by the finder needle, a 14-gauge angiocath was placed followed by passage of a number 5 FiberWire suture (Arthrex GmbH, Munich, Germany), which was retrieved through the incision using a Maryland grasper or a tendon passer. This suture was attached to a small bone plate with four holes using the two central holes. One lateral hole is used to tie an umbilical tape, which will be used to pull out the bone plate from within the chest when it is no longer needed. The FiberWire suture that exits the anterior chest wall is then tied to the Rultract Retractor (Rultract Inc., Cleveland, OH) or Easy Crank System (Primemed Inc., Sungnam City, Korea: www.pmdmdllc.com), and the chest is elevated. After the chest elevation, the anterior mediastinum was dissected bluntly using an endopeanut under thoracoscopic vision. After completion of the bilateral four-level cryoanalgesia, a metal bar (Zimmer Biomet, Jacksonville, FL) was chosen such that the ends of the bar will rest at the anterior axillary line; in other words, a short bar is utilized rather than picking a bar that will reach the mid-axillary line. On average the bar length was either 9 or 10 inches long. The ends of the bar are also kept straight to allow the stabilizer to slide toward the medial chest location. Once the introducer has passed across the chest, the bone plates used to elevate the chest are pulled out. The bar is passed across the chest with a 28 Fr chest tube and rotated into the final place. Stabilizers are applied to both sides and pulled in medially using two vein retractors applied to both wings of the stabilizer by an assistant who is facing the surgeon on the other side of the patient. The distal tip of the bar is then bent downward toward the chest wall, creating a curvature on the metal, which locks in the stabilizer (Fig. 2). Typically, 2 to 3 centimeters of the metal bar extends beyond the stabilizer notch after bending of the metal implant. To facilitate the bending of the end parts of the metal bar, two Synthes 5.0 Benders are used (Depuy Synthes, Raynham, MA). No suture fixation is necessary once the ends of the bar are bent inward. The Synthes 5.0 Bender is also useful for stabilizer/bar disengagement during a bar removal procedure. Figure 3 illustrates the methods to disengage the stabilizer from the metal implant using the Synthes Benders. Once the stabilizers are locked in, both wounds are thoroughly irrigated. No sutures are used to secure the stabilizers. Pneumothorax is evacuated, and the wounds are closed in multiple layers using absorbable sutures.

### Postoperative care and follow-up

All children were discharged to home postoperative day one with activity clearance to resume full activity (non-contact sports), which they can resume as soon as they feel their chest stiffness does not hamper their breathing. Contact sports clearance was given after seeing the patient 2 weeks after discharge during their first postoperative examination. Patients were then seen again at a 6 month interval to assess bar displacement, cosmesis and satisfaction with repair and to determine the appropriate time for bar removal, which was typically done in 2 years.

## Results

One hundred fourteen children who underwent the Nuss procedure from 2016 to 2023 were included in the study. Eight children who had moved residence prior to bar removal or not yet reached the 6 month follow-up time-point were excluded from the study. The main symptoms for most children were shortness of breath and exercise intolerance. The median age of the patient was 15 years old. Most children were male (103/114, 90%), with low BMI (Body Mass Index) (median 18.2). A few patients attempted suction device applications but could not achieve correction and decided to proceed with surgery. There were no intraoperative complications and only four children (4%) had postoperative complications, which were either hematoma formation (2) or wound infections (2), which required incision and drainage. The wound/hematomas occurred at the site of lateral chest incisions, not at the stabilizer location. These patients did not need prolonged intravenous antibiotics or need their bar removed. The average estimated blood loss for the operation was 5 ml. The average length of operative time was 130 minutes. There were no children who had bar displacement or needed surgical readjustment. There was no incidence of metal allergies. None of the patients had a metal allergy skin testing done by a dermatologist. None of the patients had pectus excavatum recurrence after the bar removal. There was no incidence of pleural effusion formation, which needed drainage or steroids. There were two cases of bar/stabilizer removal difficulties due to one end of the stabilizer having slipped under the rib. The slipped stabilizer made disengaging the stabilizer and the metal rod difficult. There were no lung injuries. The data is stratified in Table 1.

**Table 1:** Patient cohort and postoperative outcomes are shown

Category	Median (IQR)
Sex (male)	103 (90%)
Age	15 (14–17)
Previous intervention	
Brace	2 (2%)
Vacuum bell	6 (5%)
Haller index	3.9 (3.7–4.5)
BMI	18.2 (17.3–19.5)
Comorbidities	
Asthma	4 (4%)
Scoliosis	5 (4%)
Cardiac history	2 (2%)
Intraoperative complications	0
Intraoperative 4 level cryoanalgesia	114 (100%)
Postoperative complications	
Infection requiring antibiotics	2 (2%)
Hematoma requiring drainage	2 (2%)
Bar displacement	0
Length of hospital stay	1 (1–2)
Time to bar removal (months)	14 (10–24)
Complications at removal	0

## Discussion

In our patient cohort, we found that no children suffered from bar displacement when medially positioned bilateral stabilizers were utilized. No activity restrictions were imposed on the patients postoperatively; the level of activity was left up to the patient to decide. The wound infection complication rate was comparable to other techniques reported in the literature [16].

The modification that we have adopted is one of many efforts to reduce the rates of bar displacement since Nuss procedure introduction. The initial bar displacement rate was reported to be around 12% in the first decade of the Nuss procedure [1, 8, 16, 17]. Displacement rates improved to 5% with the introduction of metal stabilizers, usually placed on the left lateral side, and to 1-2% with the addition of pericostal polydioxanone (PDS) sutures [1, 7]. Other groups have advocated various stabilizing techniques, but no consensus has yet been reached over the optimal number of stabilizers or pericostal reinforcements [10–12]. Park and colleagues in Korea have systematically tested and reported on several techniques to reduce bar displacement rates. Park initially described a five-point fixation method that was associated with a decrease in displacement rates from 4.6 to 1.8% in a cohort of 725 patients [3]. Park’s group later advocated for claw fixators, hinge plates, and finally double bar placement with a metal bridge to connect the two bars together, with a reported bar displacement rate of 0% [18]. These promising single-center results point to the possibility of a prospective, multi-site study to directly compare different approaches and associated outcomes.

The aim of stabilizers, pericostal sutures, and wires is to limit the DOF of the substernal bar. Our institution’s 0% bar displacement rate illustrates that if 3 DOF are blocked by counteracting forces the bar dislocation will not occur. Lateral movement of the bar is inhibited by the resistance from bilateral stabilizers, which are locked in medially by the chest wall and laterally by the curvature of the metal bar. Rotation of the bar along the frontal axis is prevented by the anterior chest stabilizer position. Anterior/posterior translation is prevented by the bilateral stabilizers resting on two rib surfaces. It is critical that the stabilizer rests on two ribs on each side of the chest. For older patients, whose rib spaces are farther apart, an addition of bone metal plate that is longer than the current stabilizer length made by Zimmer Corporation may be prudent. Our discharge instructions encouraged early return to school and sports activity. This contrasts with typical postoperative instructions given to patients to stay at home for 2–3 weeks after bar placement, to lie in the supine position and avoid lateral pressure for 6 weeks, and to avoid sports or heavy lifting for 6 weeks. Others have instructed competitive sports could be re-initiated after 3 months, but “heavy contact sports such as boxing, football, and hockey are not permitted when the bar is in place” [1, 16]. Still others recommended limiting activity to activities of daily living for 6 weeks post-procedure, to resume sports at 3 months, and to avoid contact sports, while acknowledging that such restrictions are contestable and not evidence-based [9]. There is no evidence in the surgical literature to support a causal link between postoperative activity and bar displacement, although there may be probable causal relation when the technique utilized is ineffective in blocking the 3 DOF. Common activity restriction often used highlights the surgeon’s low confidence in the stability of the metal bar rather than supporting the argument that the level of a patient’s physical activity affects bar dislocation rate. There is no argument, however, against the potential behavioral, cognitive, and social benefits of postoperative physical activity for children, as advocated by the American Academy of Pediatrics’ recommendation of at least 60 minutes of moderate or vigorous activity per day for children 6–17 years old [19]; hence, it is incumbent upon surgeons to develop and utilize techniques that will make this happen.

The limitations of our study include the small sample size and single-center nature of the study operated on by three surgeons. Due to the retrospective nature of the study, we do not know what percentage of children participated in contact sports. In addition to the medial stabilizer configuration, using shorter bars may have had a positive effect on the stability of the metal/stabilizer unit since the rotational force on the metal bar would be less compared to a longer bar with greater curvature. Prior to our decision to place the stabilizer in the medial position, we, like others, placed the stabilizers in the lateral location with suture fixations. We decided to change our practice, even though we did not experience bar dislocations in our patients, due to the literature reports of bar dislocation in other patients. Unlike other reports of using sutures or wires to fix the bar, suture fixation is not necessary if metal components themselves are used to lock in their construction as is described in this report. One difficulty, however, that can be encountered with the medial stabilizer position is the occasional difficulty of separating the stabilizer from the metal bar at the time of the bar removal. Separation is technically easy if the thin Synthes bar benders are used to physically bend the stabilizer itself such that it disengages from the metal bar. However, this can become difficult if one end of the stabilizer slips under the resting rib. This occurred in two cases found at the time of the bar removal. There was no chest depression found in these patients due to the stabilizer slippage, however. Due to this drawback, we can state that positioning the stabilizer in the medial location is not the panacea operation that makes a surgeon’s life completely carefree. Although the method used in this study was applied to patients less than 18 years old, we suspect it should work similarly in adult patients.

## Conclusion

We found that children who underwent operative repair of pectus excavatum using medial bilateral bar stabilizers coupled with no activity restriction did not experience bar displacement. Thoughtful technical consideration should be made on how to block the implanted metal’s 3 DOF. When proper surgical techniques are applied, it would be acceptable to allow children to return promptly to physical activity without restrictions.

## Data Availability

No datasets were generated or analysed during the current study.
